# Perspective: COVID-19 and Its Neurologic Sequelae

**DOI:** 10.31480/2330-4871/162

**Published:** 2022

**Authors:** Aaron Rothstein, Christopher Favilla, Kelly Sloane, Jens Witsch

**Affiliations:** Department of Neurology, Perelman School of Medicine at the University of Pennsylvania, Philadelphia, Pennsylvania, USA

**Keywords:** COVID-19, Neurology, Stroke, SARS-CoV-2, Pandemic

## Abstract

COVID-19 led to a catastrophic, international, public health crisis after its first detection in 2019 [1]. Though it is primarily a respiratory virus, it impacts the central and peripheral nervous systems leading to further COVID-19-associated disability [2]. This Perspective reviews our current understanding of the neurological sequelae of COVID-19 and the gaps in our understanding of their treatment and epidemiology.

## Introduction

COVID-19, the viral illness caused by severe acute respiratory syndrome coronavirus 2 (SARS-CoV-2), led to a catastrophic, international, public health crisis after its first detection in 2019 [1]. The virus has infected over 250 million people worldwide and has taken the lives of over 6 million people [3,4]. Though it is primarily a respiratory virus, it impacts several organ systems, including the central and peripheral nervous systems. Neurological sequelae of the virus represent a critical contribution to COVID-19-associated disability [2].

Coronaviruses like SARS-CoV-2 lead to neurologic sequelae either directly--through viral infection-- or indirectly--via immune mediated mechanisms. Directly, viral particles can invade the nervous system via the olfactory tract thus activating macrophages, microglia and astrocytes resulting in direct cell damage or meningitis/encephalitis [5]. Indirectly, immune-mediated, post-infectious effects include pathologies such as Guillain-Barre syndrome (GBS), transverse myelitis, and optic neuritis [5,6].

A range of neurologic manifestations of SARS-CoV-2 have been reported during and after infection (Table 1 and Table 2). The prevalence of neurological disorders among all hospitalized patients with COVID-19 was 13.5%, and these included toxic-metabolic encephalopathy (51% of those with neurological disorders), stroke (14%), and seizures (12%) among others [7].

The incidence of encephalopathy in hospitalized patients with COVID-19 ranges widely from 7–69%. Although the vast majority of patients experience an improvement in mentation following medical management, COVID-19 associated encephalopathy is associated with an increased risk of mortality [8].

Stroke is one of the most commonly reported acute effects of COVID-19. At one institution, 2.4% of hospitalized patients with COVID-19 had ischemic stroke while 0.9% had intracranial hemorrhage [9]. Other studies demonstrated an ischemic stroke rate in hospitalized patients with COVID-19 of 0.5% to 1.6% [7]. Moreover, patients with COVID-19 suffer more severe strokes, with higher mortality than their non-infected counterparts (39.3% vs. 16.1%)--though this was likely subject to potential bias in early reporting [10].

Acute, symptomatic seizures are reported in fewer than 1% of patients with COVID-19, indicating that the risk of seizures is rarer than with other viral diseases such as SARS (Severe Acute Respiratory Syndrome) or MERS (Middle East Respiratory Syndrome) [11]. However, in a meta-analysis of hospitalized COVID-19 patients there was a 20.3% prevalence of epileptiform discharges on EEG, an indication of seizure potential. COVID-19 may also affect the seizure frequency of those with underlying seizure disorders but the extent to which it does remains uncertain [8,12].

These acute and severe neurological manifestations of COVID-19 clearly impact clinical outcomes and prognosis. In a study of hospitalized patients with COVID-19, in-hospital mortality was 15.3% in patients who presented with COVID-19 and associated neurological manifestations. Moreover, patients with severe neurological manifestations, including encephalopathy or stroke, experienced a mortality rate of 37.4% compared to 11.9% in those who had minor neurologic manifestations during infection [7]. The relationship between acute COVID-19 associated neurological symptoms and mortality, in addition to respiratory failure and ICU admission, was confirmed in other reports [13]. However, sepsis from other infections with concomitant neurologic symptoms portends a poorer prognosis, too [14]. Thus, it is not clear how specific this connection between neurologic symptoms and poor prognosis is to COVID-19 compared to other infectious sources.

In addition to the neurological manifestations seen during acute COVID-19 infection, some patients develop less severe, yet distressing, neurologic sequelae from the virus which can last beyond the infectious period. Headache is one of the most common neurologic manifestations in COVID-19, reported in nearly half of patients with the virus [5,15]. Olfactory and gustatory dysfunction as well as cognitive and mental health difficulties have also been reported [16]. Anosmia and hyposmia were reported by 48.23% of COVID-19 patients in one study while 83.38% had decreased taste sensation within 4 weeks from the onset of infection [15]. The incidence of anosmia or gustatory dysfunction also varies depending on the circulating variant. Indeed, the reported risk of olfactory dysfunction ranges from 5–98% [5]. Most patients do recover these senses partially if not wholly over the course of weeks [17]. Cognitive symptoms are common and widely documented, too. In patients with symptoms 4 months after COVID-19 infection, 67% demonstrated 1 or more abnormally low cognitive scores, with processing speed being the most common deficit [18]. In a prospective case-control study, cognitive status using the MoCA (Montreal Cognitive Assessment) at 6 months was worse among survivors of COVID-19 than age, sex, and ICU status-matched controls [19].

Many COVID-19-associated neurological symptoms persist long after the respiratory process passes. One year after COVID-19 infection, persistent neurological sequelae were reported in 12% of patients; symptoms included fatigue, concentration difficulties, forgetfulness, sleep disturbances, limb weakness, and headache in descending order of prevalence [20]. A year following severe COVID-19, 87% of patients had ongoing abnormalities in functional, cognitive, or neurological quality of life metrics [21]. Nevertheless, the natural history of COVID-19 neurological sequelae is still being written and is further complicated by subjective symptom reporting [22].

## Conclusion

Neurological manifestations of COVID-19, both direct and indirect, are critical to the clinical impact of the virus. Patients who contract the virus, particularly those that are hospitalized, have an increased risk of stroke, encephalopathy, headache, dizziness, loss of smell and taste, and cognitive sequelae. And while the quality of the studies pointing to these risks vary based on their methodology and design, there seems to be some connection between COVID-19 and neurological symptoms. Moreover, those who develop neurological symptoms or have an underlying neurological disease have an increased risk of poor outcomes, including death [7]. Furthermore, the COVID-19 pandemic significantly strained systems of acute neurologic care- and such impacts extend beyond the peak period of the pandemic [23–25]. Potential solutions to reduce this impact have been proposed, but their effectiveness has yet to be demonstrated [26–28]. Thus, while we collect more epidemiological data to parse out quantitative risk for symptoms and outcomes in the population, we ought to look for proven methods to mitigate that risk and the resultant adverse outcomes.

Indeed, there is much that is required from clinical research to improve neurological outcomes in COVID-19 patients. We need further investigation into the relationship between COVID-19 and neurology. But we also need to understand how to treat neurological sequelae and how our approach to COVID-19, our antiviral therapies, and even our vaccines, affect neurological outcomes. This presents the next challenge for neurology patients in the fight against COVID-19.

## Figures and Tables

**Table 1:** Pooled prevalence of select neurological sequelae during COVID-19 infection based on meta-analyses.

Neurological Symptoms/Diagnoses	Pooled Prevalence
Seizures	1–4.05% [29,30]
Encephalopathy	7–23.5% [29,30]
Ischemic stroke	1.3–3% [31–33]
Hemorrhagic stroke	0.4–0.6% [32,33]
Olfactory dysfunction	19–55% [29,30,34]
Gustatory dysfunction	21–41% [29,30,34,35]
Headache	13–47.1% [29,30,36,37]
Fatigue	32–37.8% [29,30,36]
Peripheral Neuropathy	1% [30]
Cognitive impairment	2% [30]

**Table 2:** Pooled prevalence of select neurological sequelae after COVID-19 infection based on meta-analyses.

Neurological Symptoms/Diagnoses	Pooled Prevalence
Sleep disturbance	11–27.4% [38–40]
Olfactory dysfunction	7–15.1% [39,40]
Dizziness	2.9–9.7% [38,39]
Headache	6.6–44% [37,38,41]
Fatigue	23–58% [38–42]
Cognitive impairment	14–22% [38–40,42]

## References

[1] Akkız H The Biological Functions and Clinical Significance of SARS-CoV-2 Variants of Corcern. Front Med. 2022;9: 849217. doi:10.3389/fmed.2022.849217 PMC9163346 35669924

[2] Whittaker A, Anson M, Harky A. Neurological Manifestations of COVID-19: A systematic review and current update. Acta Neurol Scand. 2020;142: 14–22. doi:10.1111/ANE.13266 32412088 PMC7273036

[3] Tannous J, Vahidy FS. The Collateral Damage of COVID-19. Neurology. 2022;98: 219–220. doi:10.1212/WNL.0000000000013196 35131916

[4] WHO Coronavirus (COVID-19) Dashboard | WHO Coronavirus (COVID-19) Dashboard With Vaccination Data. [cited 28 Jul 2022]. Available: https://covid19.who.int/

[5] Dewanjee S, Vallamkondu J, Kalra RS, Puvvada N, Kandimalla R, Reddy PH. Emerging COVID-19 Neurological Manifestations: Present Outlook and Potential Neurological Challenges in COVID-19 Pandemic. Mol Neurobiol. 2021;58: 4694–4715. doi:10.1007/S12035-021-02450-6 34169443 PMC8224263

[6] Sriwastava S, Tandon M, Podury S, Prasad A, Wen S, Guthrie G, COVID-19 and neuroinflammation: a literature review of relevant neuroimaging and CSF markers in central nervous system inflammatory disorders from SARS-COV2. J Neurol. 2021;268: 4448–4478. doi:10.1007/S00415-021-10611-9 34009454 PMC8131883

[7] Beghi E, Giussani G, Westenberg E, Allegri R, Garcia-Azorin D, Guekht A, Acute and post-acute neurological manifestations of COVID-19: present findings, critical appraisal, and future directions. J Neurol. 2022;269: 2265–2274. doi:10.1007/S00415-021-10848-4 34674005 PMC8528941

[8] Ahmad SJ, Feigen CM, Vazquez JP, Kobets AJ, Altschul DJ. Neurological Sequelae of COVID-19. J Integr Neurosci. 2022;21. doi:10.31083/J.JIN2103077 35633158

[9] Rothstein A, Oldridge O, Schwennesen H, Do D, Cucchiara BL. Acute Cerebrovascular Events in Hospitalized COVID-19 Patients. Stroke. 2020;51: e219–e222. doi:10.1161/STROKEAHA.120.030995 32684145 PMC7386677

[10] Martí-Fàbregas J, Guisado-Alonso D, Delgado-Mederos R, Martínez-Domeño A, Prats-Sánchez L, Guasch-Jiménez M, Impact of COVID-19 Infection on the Outcome of Patients With Ischemic Stroke. Stroke. 2021;52: 3908–3917. doi:10.1161/STROKEAHA.121.034883 34455823 PMC8607902

[11] Kuroda N. Epilepsy and COVID-19: Updated evidence and narrative review. Epilepsy Behav. 2021;116: 107785. doi:10.1016/J.YEBEH.2021.107785 33515934 PMC7805398

[12] Kuroda N, Gajera PK, Yu H, Kubota T. Seizure Control in Patients with Epilepsy During the COVID-19 Pandemic: A Systematic Review and Meta-analysis. Intern Med. 2022 [cited 29 Jul 2022]. doi:10.2169/INTERNALMEDICINE.9321-22 PMC9424088 35650127

[13] Espiritu AI, Sy MCC, Anlacan VMM, Jamora RDG. COVID-19 outcomes of 10,881 patients: retrospective study of neurological symptoms and associated manifestations (Philippine CORONA Study). J Neural Transm. 2021;128: 1687–1703. doi:10.1007/S00702-021-02400-5 34448930 PMC8391861

[14] Hocker SE, Wijdicks EFM. Neurologic complications of sepsis. Continuum (Minneap Minn). 2014;20: 598–613. doi:10.1212/01.CON.0000450968.53581.FF 24893236 PMC10563989

[15] Hensley MK, Markantone D, Prescott HC. Neurologic Manifestations and Complications of COVID-19. https://doi.org/101146/annurev-med-042320-010427. 2022;73: 113–127. doi:10.1146/ANNUREV-MED-042320-010427 34416121

[16] Cousyn L, Sellem B, Palich R, Bendetowicz D, Agher R, Delorme C, Olfactory and gustatory dysfunctions in COVID-19 outpatients: A prospective cohort study. Infect Dis now. 2021;51: 440–444. doi:10.1016/J.IDNOW.2021.03.004 33766735 PMC7983360

[17] Teaima AA, Salem OM, Teama MAEM, Mansour OI, Taha MS, Badr FM, Patterns and clinical outcomes of olfactory and gustatory disorders in six months: Prospective study of 1031 COVID-19 patients. Am J Otolaryngol. 2022;43. doi:10.1016/J.AMJOTO.2021.103259 PMC8481118 34626912

[18] Vannorsdall TD, Brigham E, Fawzy A, Raju S, Gorgone A, Pletnikova A, Cognitive Dysfunction, Psychiatric Distress, and Functional Decline After COVID-19. J Acad Consult Psychiatry. 2022;63: 133–143. doi:10.1016/J.JACLP.2021.10.006 PMC8591857 34793996

[19] Nersesjan V, Fonsmark L, Christensen RHB, Amiri M, Merie C, Lebech AM, Neuropsychiatric and Cognitive Outcomes in Patients 6 Months After COVID-19 Requiring Hospitalization Compared With Matched Control Patients Hospitalized for Non-COVID-19 Illness. JAMA psychiatry. 2022;79: 486–497. doi:10.1001/JAMAPSYCHIATRY.2022.0284 35319743 PMC8943626

[20] Rass V, Beer R, Schiefecker AJ, Lindner A, Kofler M, Ianosi BA, Neurological outcomes 1 year after COVID-19 diagnosis: A prospective longitudinal cohort study. Eur J Neurol. 2022;29: 1685–1696. doi:10.1111/ENE.15307 35239247 PMC9111823

[21] Frontera JA, Yang D, Medicherla C, Baskharoun S, Bauman K, Bell L, Trajectories of Neurologic Recovery 12 Months After Hospitalization for COVID-19: A Prospective Longitudinal Study. Neurology. 2022;99: 10.1212/WNL.0000000000200356. doi:10.1212/WNL.0000000000200356 PMC9259089 35314503

[22] Matta J, Wiernik E, Robineau O, Carrat F, Touvier M, Severi G, Association of Self-reported COVID-19 Infection and SARS-CoV-2 Serology Test Results With Persistent Physical Symptoms Among French Adults During the COVID-19 Pandemic. JAMA Intern Med. 2022;182: 19–25. doi:10.1001/JAMAINTERNMED.2021.6454 34747982 PMC8576624

[23] Zhao J, Wang Y, Fisher M, Liu R. Slower recovery of outpatient clinics than inpatient services for stroke and other neurological diseases after COVID-19 Pandemic. CNS Neurosci Ther. 2020;26: 1322–1326. doi:10.1111/CNS.13459 33058536 PMC7675482

[24] Liu R, Zhao J, Fisher M. The global impact of COVID-19 on acute stroke care. CNS Neurosci Ther. 2020;26: 1103–1105. doi:10.1111/CNS.13442 32725844 PMC7539838

[25] Nogueira RG, Qureshi MM, Abdalkader M, Martins SO, Yamagami H, Qiu Z, Global Impact of COVID-19 on Stroke Care and IV Thrombolysis. Neurology. 2021;96: e2824–e2838. doi:10.1212/WNL.0000000000011885 33766997 PMC8205458

[26] Zhao J, Li H, Kung D, Fisher M, Shen Y, Liu R. Impact of the COVID-19 Epidemic on Stroke Care and Potential Solutions. Stroke. 2020;51: 1996–2001. doi:10.1161/STROKEAHA.120.030225 32432997 PMC7258753

[27] Liu R, Fisher M, Rudd A, Zhao J. Speech disturbance plays critical role in stroke recognition during COVID-19 pandemic. CNS Neurosci Ther. 2021;27: 267–269. doi:10.1111/CNS.13608 33452869 PMC7871789

[28] Zhao J, Rudd A, Liu R. Challenges and Potential Solutions of Stroke Care During the Coronavirus Disease 2019 (COVID-19) Outbreak. Stroke. 2020;51: 1356–1357. doi:10.1161/STROKEAHA.120.029701 32228369 PMC7219852

[29] Vitalakumar D, Sharma A, Kumar A, Flora SJS. Neurological Manifestations in COVID-19 Patients: A Meta-Analysis. ACS Chem Neurosci. 2021;12: 2776–2797. doi:10.1021/ACSCHEMNEURO.1C00353/SUPPL_FILE/CN1C00353_SI_001.PDF 34260855

[30] Misra S, Kolappa K, Prasad M, Radhakrishnan D, Thakur KT, Solomon T, Frequency of Neurologic Manifestations in COVID-19 A Systematic Review and Meta-analysis. 2021. doi:10.1212/WNL.0000000000012930 PMC8665434 34635561

[31] Luo W, Liu X, Bao K, Huang C. Ischemic stroke associated with COVID-19: a systematic review and meta-analysis. J Neurol. 2022;269: 1731–1740. doi:10.1007/S00415-021-10837-7 34652503 PMC8517946

[32] Huth SF, Cho SM, Robba C, Highton D, Battaglini D, Bellapart J, Neurological Manifestations of Coronavirus Disease 2019: A Comprehensive Review and Meta-Analysis of the First 6 Months of Pandemic Reporting. Front Neurol. 2021;12. doi:10.3389/FNEUR.2021.664599 PMC8387564 34456840

[33] Ganesh A, Reis IR, Varma M, Patry DG, Cooke LJ. Neurological and Head/Eyes/Ears/Nose/Throat Manifestations of COVID-19: A Systematic Review and Meta-Analysis. Can J Neurol Sci. 2021; 1. doi:10.1017/CJN.2021.180 PMC8460425 34287109

[34] Esmaeili M, Abdi F, Shafiee G, Asayesh H, Abdar ZE, Baygi F, Olfactory and Gustatory Dysfunction in 2019 Novel Coronavirus: An Updated Systematic Review and Meta-analysis. Int J Prev Med. 2021;12. doi:10.4103/IJPVM.IJPVM_484_20 PMC8724794 35070203

[35] Hannum ME, Koch RJ, Ramirez VA, Marks SS, Toskala AK, Herriman RD, Taste loss as a distinct symptom of COVID-19: a systematic review and meta-analysis. Chem Senses. 2022;47. doi:10.1093/CHEMSE/BJAC001 PMC8849313 35171979

[36] Rogers JP, Watson CJ, Badenoch J, Cross B, Butler M, Song J, Neurology and neuropsychiatry of COVID-19: a systematic review and meta-analysis of the early literature reveals frequent CNS manifestations and key emerging narratives. J Neurol Neurosurg Psychiatry. 2021;92: 932–941. doi:10.1136/jnnp-2021-326405 34083395

[37] Fernández-de-las-Peñas C, Navarro-Santana M, Gómez-Mayordomo V, Cuadrado ML, García-Azorín D, Arendt-Nielsen L, Headache as an acute and post-COVID-19 symptom in COVID-19 survivors: A meta-analysis of the current literature. Eur J Neurol. 2021;28: 3820–3825. doi:10.1111/ENE.15040 34327787 PMC8444899

[38] Badenoch JB, Rengasamy ER, Watson C, Jansen K, Chakraborty S, Sundaram RD, Persistent neuropsychiatric symptoms after COVID-19: a systematic review and meta-analysis. Brain Commun. 2021;4. doi:10.1093/BRAINCOMMS/FCAB297 PMC8833580 35169700

[39] Zeng N, Zhao Y-M, Yan W, Li C, Lu Q-D, Liu L, A systematic review and meta-analysis of long term physical and mental sequelae of COVID-19 pandemic: call for research priority and action. doi:10.1038/s41380-022-01614-7 PMC9168643 35668159

[40] Chen C, Haupert SR, Zimmermann L, Shi X, Fritsche LG, Mukherjee B. Global Prevalence of Post COVID-19 Condition or Long COVID: A Meta-Analysis and Systematic Review. J Infect Dis. 2022 [cited 29 Jul 2022]. doi:10.1093/INFDIS/JIAC136 PMC9047189 35429399

[41] Lopez-Leon S, Wegman-Ostrosky T, Perelman C, Sepulveda R, Rebolledo PA, Cuapio A, More than 50 long-term effects of COVID-19: a systematic review and meta-analysis. Sci Reports |. 123AD;11: 16144. doi:10.1038/s41598-021-95565-8 PMC8352980 34373540

[42] Ceban F, Ling S, Lui LMW, Lee Y, Gill H, Teopiz KM, Fatigue and cognitive impairment in Post-COVID-19 Syndrome: A systematic review and meta-analysis. Brain Behav Immun. 2022;101: 93–135. doi:10.1016/J.BBI.2021.12.020 34973396 PMC8715665

